# Short-chain fatty acids play a positive role in colorectal cancer

**DOI:** 10.1007/s12672-024-01313-5

**Published:** 2024-09-10

**Authors:** Gang Liu, Jingtong Tang, Jianping Zhou, Ming Dong

**Affiliations:** 1 Department of Gastrointestinal Surgery & Hernia and Abdominal Wall Surgery, The First Hospital of China Medical University, Shenyang, 110001 Liaoning China; 2 Shenyang Medical Nutrition Clinical Medical Research Center, Shenyang, 110001 Liaoning China

**Keywords:** Short-chain fatty acids, Colorectal cancer, Research progress

## Abstract

Short-chain fatty acids (SCFAs) are produced by bacterial fermentation in the colon and are thought to be protective against gastrointestinal disease. SCFAs such as acetate, propionate and butyrate are important metabolites in the maintenance of intestinal homeostasis and have been shown to be beneficial in colorectal cancer (CRC). SCFAs are responsible for maintaining a normal intestinal barrier and exhibit numerous immunomodulatory functions. In this review article, we will discuss the metabolism and mechanism of action of SCFAs and their effects on the CRC, with particular emphasis on dietary fiber treatment and the clinical research progress.

## Introduction

Short-chain fatty acids (SCFAs) are substances released during the bacterial fermentation of dietary fiber in the gut [1, 2]. In the human body, the most abundant SCFA (≥ 95%) are acetate, propionate and butyrate; their molar ratio is approximately 6:2:2 [3]. Other SCFAs such as formate, valerate and caproate are present in the body in much smaller amounts. Small amounts of SCFA are obtained directly from food, but their main source is fermentation of dietary fiber in the colon [4]. Each day, about 500–600 mmol SCFA is produced in the intestine, however, this value is dependent on many factors, such as the amount of fiber supplied, intestinal transit time, and the composition of the intestinal microbiota. The biological functions of SCFAs include reducing the pH of colon to inhibit the growth of destructive bacteria, and regulating energy metabolism, inflammation, and tumor growth and development [5]. Besides, SCFAs also aid in managing immune regulation, appetite regulation, lipid metabolism and glucose metabolism [6, 7].

## Relationship between SCFAs and CRC

Colorectal cancer (CRC) is the 3rd most commonly diagnosed cancer (10.0% of the total cases) and the second leading cause of cancer-related mortality (9.4% of the total cancer deaths) in the world [8]. Each year, about 1–2 million new cases of CRC are reported, and 600,000 people die from it. CRC is the most closely related to diet among all cancer types, with 30–50% of colorectal cancer patients being related to diet and nutrition [9]. A large number of epidemiological data and clinical trials have proved that red meat such as beef, pork, and mutton, and processed meat such as sausage and bacon that have been cured and smoked can significantly increase the risk of colorectal cancer [10, 11]. This is an important factor in the increasing incidence of colorectal cancer worldwide. The carcinogenicity of red meat and processed meat may be related to some carcinogens contained in it, and the specific mechanism is not clear. Numerous studies have shown that people who consume more dietary fiber have a relatively low incidence of colorectal cancer, and intake of 10 g of dietary fiber per day can reduce the risk of colorectal cancer by 10%, which is inseparable from the role of SCFAs [12–14].

SCFAs are produced by the beneficial bacteria in the microbiome, and they are essential for gut and brain. Butyrate, propionate and acetate are the most abundant SCFAs in the human body. SCFAs significantly improve the function of the intestines, including taking part in maintaining the integrity of the intestinal barrier, protecting against inflammation, increasing mucus production, and stimulating intestinal motility [15–17]. Numerous studies suggest their protective and pro-health activity in pathologies of the gastrointestinal tract, such as inflammatory bowel diseases (IBD) and CRC [18–21].

Butyrate, one of the most important SCFAs, was produced by healthy gut microbiota (including *Coprococcus comes, Coprococcus eutactus, Anaerostipes spp., Coprococcus catus, Eubacterium rectale, Eubacterium hallii, Faecalibacterium prausnitzii,Roseburia spp.*) [22]. Lactate and acetate may serve as substrates for the production of butyrate [23]. Recent studies have shown that low levels of butyrate are associated with a higher incidence of CRC [24, 25]. In fact, butyrate is foremost source of energy supply and also stimulates mucosal proliferation under certain conditions. When epithelial cells are energy-deficient, butyrate is used for energy supply; when energy is sufficient, butyrate is used to induce DNA-damaged cell differentiation, apoptosis, and inhibit tumor cell proliferation [26, 27]. Ma X [28] also showed that butyrate significantly inhibits liver metastasis of CRC cells, improves intestinal dysbiosis in mice and enhances antitumor immune responses in liver of mice. Although acetate and propionate are not as powerful as butyrate in preventing CRC, they still show good protection in a large number of studies (Table 1).

**Table 1:** Relationship of SCFAs in CRC

SCFAs	Type of study	Species	Results	Mechanism	References
Butyrate	In vivo	Mice	↑Apoptosis of CRC cells ↑Differentiation of CRC cells ↓liver metastasis of CRC cells	–	Ma et al. [28]
	In vitro	Human	↑Apoptosis of CRC cells	Suppressing promoter methylation of the proapoptotic genes Bcl2l11, Cideb, Dapk1, Ltbr, and Tnfrsf25	Cho et al. [29]
	In vivo	Rats	↑Apoptosis of CRC cells ↓Proliferation of CRC cells ↑Protection against CRC	–	Hong et al. [30]
	In vitro	Human	↑Apoptosis of CRC cells ↓G2-M cycle	Increasing the level of intracellular ROS	Matthews et al. [31]
	In vitro	Human	↑Apoptosis of CRC cells	Regulating target genes, including cell cycle-related *EIF4G2* and *BIRC5*	Ali et al. [32]
	In vitro	Human	↑Differentiation of CRC cells	Increaseing production of ATP by oxidative phosphorylation	Klepinina et al. [33]
	In vitro	Human	↑Apoptosis of CRC cells ↓Proliferation of CRC cells	Decreasing c-Myc and miR-92a levels and increasing p57 expression	Hu et al. [34]
	In vivo	Mice	↓Tumor proliferation	suppressing transcription factors including SREBP and enzymes critical for cholesterol synthesis	Broadfield et al. [35]
	In vivo	Mice	↑CRC cells death	Through the butyrate/OR51E1/RALB axis	Dong et al. [36]
Propionate	In vitro	Human	↑Apoptosis of CRC cells ↑autophagy of CRC cells	Decreasing mTOR activity and enhanced AMP kinase activity	Tang et al. [37]
	In vitro	Human	↓Proliferation of CRC cells ↑Apoptosis of CRC cells	Decreasing the protein stability of EHMT2 by up-regulation of HECTD2	Ryu et al. [38]
	In vitro	Human	↓Proliferation of CRC cells ↑CRC cells death	–	Casanova et al. [39]
	In vitro	Human	↑Apoptosis of CRC cells	Reducing PRMT1 level and affecting the mTOR pathway	Ryu et al. [40]
Acetate	In vitro	Human	↓Proliferation of CRC cells	Inducing lysosomal membrane permeabilization and releasing cathepsin D to the cytosol in cell	Marques et al. [41]
	In vitro	Human	↑Apoptosis of CRC cells	Accumulating of reactive oxygen species and changing in mitochondrial mass and mitochondrial membrane potential	Oliveira et al. [42]
	In vitro	Human	No effect on CRC cells growth	–	Sara et al. [43]
	In vivo	Mice	↑Tumor growth	Targeting inhibition of Acss2/HIF-2 signaling	Garcia et al. [44]

## The metabolism and mechanism of SCFAs

The gut microbiota produces SCFAs through fermentation of dietary fiber in colon. The concentration of SCFAs varies according to the section of the colon, which reaches a concentration of approximately 70–140 mM in the proximal part of the colon, and drops to 20–70 mM in the distal part of the colon. The higher concentration of SCFAs in the proximal colon is due to the greater availability of carbohydrates and water in this part of the intestine. The difference in the concentration of SCFAs means that the pH value is different along the human colon [4].

**Fig. 1:**
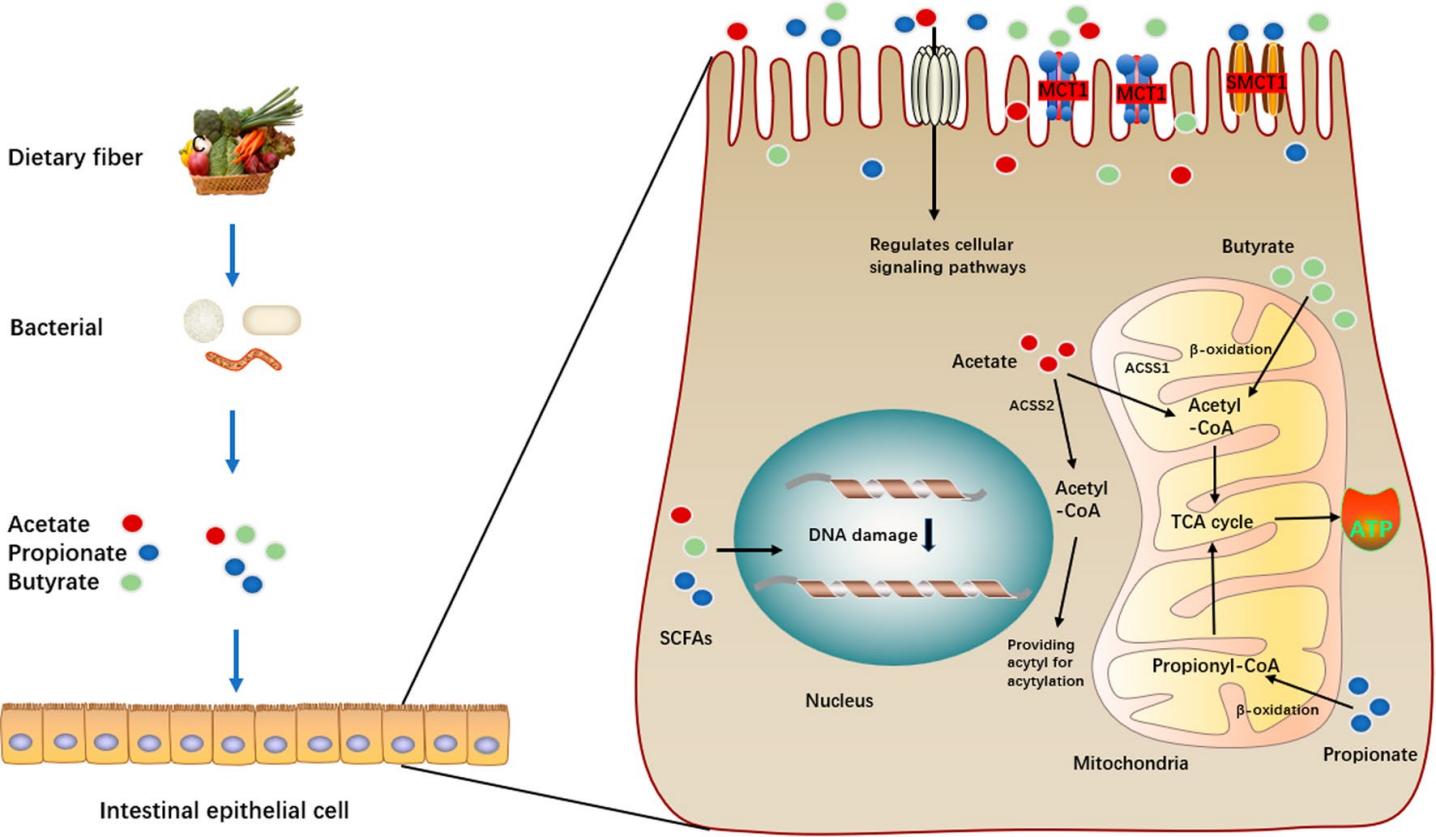
Mechanisms of SCFA action within intestinal epithelial cells. *SCFA* short-chain fatty acids, *MCT1* monocarboxylate transporter 1, *SMCT1* sodium-coupled monocarboxylate transporter 1, *ACSS1* acyl-CoA synthetase short-chain family member 1, *ACSS2* acyl-CoA synthetase short-chain family member 2, *ATP* Adenosine triphosphate

SCFAs are absorbed by colonocytes, mainly through the hydrogen and sodium dependent monocarboxylate transporters (MCTs and SMCTs) and by passive diffusion [45]. Tissues have different subtypes and patterns of MCT expression—proton-coupled monocarboxylate transporter 1 (MCT1/SLC16A1) and sodium-coupled monocarboxylate transporter (SMCT1/SLC5A8) [46]. SCFAs are rapidly absorbed by colonocytes via MCT1 and SMCT1, passively diffused or exchanged with bicarbonate (HCO3^−^) via exchangers of unknown identity, and then partially oxidized to CO_2_, producing energy for the cell in the form of ATP. CD147 is the chaperone (ancillary protein) for MCT1 [46]. Meanwhile, SCFAs can engage G-protein-coupled receptors (GPCRs) on the surface of cells regulating intracellular signaling pathways [13]. Acetate is produced from pyruvate via acetyl-CoA and it is also used to produce butyrate via Butyryl-CoA [47]. Propionate is produced from phosphoenolpyruvate via the acrylate and succinate pathways [23]. These SCFAs participate in the tricarboxylic acid cycle(TCA cycle) and generate ATP in the mitochondria of intestinal epithelial cells (Fig. 1) [48]. Intracellular actions of SCFA in colonic epithelium involve inhibition of histone deacetylases (HDACs), generation of energy, and conversion into ketone bodies [46]. Clostridium butyricum, one of the most commonly observed SCFAs producing probiotics, can inhibit the Wnt/β-catenin signaling pathway by inhibiting HDACs activity, and thus prevent the development of intestinal tumors in a murine model [13]. HDACs activity also be inhibited by butyrate to induces G1 cell cycle arrest and differentiation of human colon carcinoma cells by upregulating the negative cell cycle regulator p21Waf1/Cip1 [49].

SCFAs that are not metabolized by colonocytes enter the portal circulation of the liver through the basolateral membrane and provide energy substrates for hepatocytes through oxidation [50]. Only a small amount of acetate, propionate and butyrate reach the systemic circulation and other tissues such as skeletal muscle and adipose tissue. Recent studies on SCFAs have used fecal determination to reflect colon production of SCFAs [51–53]. In vitro experiments, Zuo et al. [54] found butyrate suppresses proliferation and migration of RKO colon cancer cells though regulating endocan expression by MAPK signaling pathway. In short, SCFAs plays a huge role in preventing the occurrence and development of CRC.

The molecular mechanism of SCFAs in CRC progression is a complex process. The gut microbiome plays a crucial role in the development of CRC by disrupting the homeostasis of the microenvironment and altering immune responses. Dysfunction of the gut microbiota can promote the occurrence of colorectal cancer, and SCFAs, as metabolites of the gut microbiota, may play a key role in this process. As an energy substrate for colon cells, SCFAs have anti-inflammatory and anticancer properties [6]. In patients with colitis, butyrate induces the release of IL-18 from colon epithelial cells by activating GPR109A, thereby participating in the regulation of colitis and colon cancer [55]. SCFAs also protect intestinal health by inducing autophagy in colon cancer cell lines [37, 56].

T cells play a crucial role in maintaining a stable intestinal environment, and SCFAs directly or indirectly regulate T cell differentiation and participate in specific cellular immunity [57]. SCFAs can alleviate intestinal inflammation by inhibiting HDACs and regulating the mTOR S6K pathway to induce the production of effector T cells and regulatory T cells [58]. So far, butyrate has been shown to prevent colitis and colon cancer under low fiber diet conditions, affecting the function of colorectal cancer cells, including regulating gene expression [59], cell signal transduction [54], and inhibiting the growth of colon cancer cells [28, 35]. The molecular mechanisms underlying the effects of propionate and acetate on colorectal cancer are also summarized in Table 1.

## Dietary fiber and SCFAs supplementation

Most nutritionists and physicians believe that a balanced diet can maximize the resistance and prevention of digestive diseases and malignant tumors. Nowadays, many young people have an imbalanced high-fat, high-meat, low-fibre diet, the proinflammatory and proneoplastic properties of protein fermentation and bile acid deconjugated residues predominate, leading to increased colon cancer risk, so colorectal cancer has a younger trend [16]. High-fibre diet is thought to provide a variety of health benefits. In addition to increasing the speed of fecal bulking and transport along the colon, fiber also provides a wide range of phytochemicals and metabolites transformed by bacteria in the human colon, of which the most important fermentation product is SCFA. A review showed high-fibre diet, in particular cereal fibre and whole grains, associated with approximately 10% lowered risk of developing CRC [12]. A prospective cohort study also demonstrated increasing fiber consumption after CRC diagnosis has been associated with better survival rates [60].

In order to provide sufficient SCFAs to the body, according to the "Chinese Residents' Dietary Nutrient Reference Intakes (2021 Edition)", adults should consume more than 25 g of dietary fiber per day. European Food Safety Authority adviced a daily intake of fiber between 25 and 32 g/d for adult women and 30–35 g/d for adult men, and for children and older adults 3–4 g/d approximately [61]. There are now two ways to consume dietary fiber. First of all, through dietary intake, such as peas, sugar beets, chicory, garlic asparagus, banana, corn, wheat, tapioca cereals, etc., but it is actually difficult to meet the demand. The second is choose products containing dietary fiber directly, such as inulin, polysaccharide, resistant dextrin or starch, etc. In fact, while engaging in dietary fiber intake, a low-fat diet is also necessary. Bile acid (BA) concentrations can reach 1 mM in the colon after the consumption of a high-fat meal, and these BAs, mostly secondary BAs in humans, are believed to be promoters of colon cancer [62]. The importance of a balanced diet deserves our attention.

## Conclusion

Although studies on the effects of SCFAs seem to show that supplements have generally positive effects on CRC, in order to obtain maximum efficacy, efforts should be made to carry out high-quality randomized controlled trials to determine the mechanism of action, the best timing, dosage, source, extraction, preparation and quantification of these products, as well as very suitable nutrition questionnaires. This will enable us to set the use of these compounds in clinical guidelines for cancer prevention.

## Data Availability

No datasets were generated or analysed during the current study.
